# Cost-effectiveness of treating advanced melanoma with tumor-infiltrating lymphocytes based on an international randomized phase 3 clinical trial

**DOI:** 10.1136/jitc-2023-008372

**Published:** 2024-03-26

**Authors:** Renske M T ten Ham, Maartje W Rohaan, Inge Jedema, Rob Kessels, Wim Stegeman, Walter Scheepmaker, Bastiaan Nuijen, Cynthia Nijenhuis, Melanie Lindenberg, Troels Holz Borch, Tine Monberg, Marco Donia, Inge Marie Svane, Wim van Harten, John Haanen, Valesca P Retel

**Affiliations:** 1 Department of Epidemiology & Health Economics, Julius Center for Health Sciences and Primary Care, Utrecht, The Netherlands; 2 Division of Psychosocial Research and Epidemiology, Netherlands Cancer Institute, Amsterdam, The Netherlands; 3 Division of Medical Oncology, Netherlands Cancer Institute, Amsterdam, The Netherlands; 4 Division of Molecular Oncology and Immunology, Netherlands Cancer Institute, Amsterdam, The Netherlands; 5 Department of Biometrics, Netherlands Cancer Institute, Amsterdam, The Netherlands; 6 Financial Department, Netherlands Cancer Institute, Amsterdam, The Netherlands; 7 Division of Pharmacy & Pharmacology, Netherlands Cancer Institute, Amsterdam, The Netherlands; 8 Biotherapeutics Unit, Netherlands Cancer Institute, Amsterdam, The Netherlands; 9 Department of Oncology, National Center for Cancer Immune Therapy, Copenhagen University Hospital, Herlev, Denmark; 10 Department of Health Technology and Services Research, University of Twente, Enschede, The Netherlands; 11 Department of Clinical Oncology, Leiden University Medical Center, Leiden, The Netherlands; 12 Erasmus School of Health Policy and Management, Erasmus University Rotterdam, Rotterdam, The Netherlands

**Keywords:** Ipilimumab, Melanoma, Healthcare Economics and Organizations

## Abstract

**Introduction:**

In a multicenter, open-label randomized phase 3 clinical trial conducted in the Netherlands and Denmark, treatment with ex vivo-expanded tumor-infiltrating lymphocytes (TIL-NKI/CCIT) from autologous melanoma tumor compared with ipilimumab improved progression-free survival in patients with unresectable stage IIIC–IV melanoma after failure of first-line or second-line treatment. Based on this trial, we conducted a cost-utility analysis.

**Methods:**

A Markov decision model was constructed to estimate expected costs (expressed in 2021€) and outcomes (quality-adjusted life years (QALYs)) of TIL-NKI/CCIT versus ipilimumab in the Netherlands. The Danish setting was assessed in a scenario analysis. A modified societal perspective was applied over a lifetime horizon. TIL-NKI/CCIT production costs were estimated via activity-based costing. Through sensitivity analyses, uncertainties and their impact on the incremental cost-effectiveness ratio (ICER) were assessed.

**Results:**

Mean total undiscounted lifetime benefits were 4.47 life years (LYs) and 3.52 QALYs for TIL-NKI/CCIT and 3.33 LYs and 2.46 QALYs for ipilimumab. Total lifetime undiscounted costs in the Netherlands were €347,168 for TIL-NKI/CCIT (including €67,547 for production costs) compared with €433,634 for ipilimumab. Undiscounted lifetime cost in the Danish scenario were €337,309 and €436,135, respectively. This resulted in a dominant situation for TIL-NKI/CCIT compared with ipilimumab in both countries, meaning incremental QALYs were gained at lower costs. Survival probabilities, and utility in progressive disease affected the ICER most.

**Conclusion:**

Based on the data of a randomized phase 3 trial, treatment with TIL-NKI/CCIT in patients with unresectable stage IIIC–IV melanoma is cost-effective and cost-saving, both in the current Dutch and Danish setting. These findings led to inclusion of TIL-NKI/CCIT as insured care and treatment guidelines. Publicly funded development of the TIL-NKI/CCIT cell therapy shows realistic promise to further explore development of effective personalized treatment while warranting economic sustainability of healthcare systems.

**WHAT IS ALREADY KNOWN ON THIS TOPIC**

- This study is the first to assess cost-effectiveness in a multicenter, open-label randomized phase 3 clinical trial of an ex vivo-expanded tumor-infiltrating lymphocytes (TIL-NKI/CCIT) from autologous melanoma tumor compared with ipilimumab in patients with unresectable stage IIIC–IV melanoma after failed first-line or second-line treatment.

**WHAT THIS STUDY ADDS**

- Treatment with TIL-NKI/CCIT in patients with unresectable stage IIIC–IV melanoma is cost-effective and cost-saving, both in the current Dutch and Danish setting. These findings led to inclusion of TIL-NKI/CCIT as insured care and treatment guidelines.

**HOW THIS STUDY MIGHT AFFECT RESEARCH, PRACTICE OR POLICY**

- Publicly funded development of the TIL-NKI/CCIT cell therapy shows realistic promise to further explore development of effective personalized treatment while warranting economic sustainability of healthcare systems.

## Introduction

Advanced melanoma is an aggressive malignant disease with high mortality as a hallmark. The introduction of targeted therapies and immune checkpoint inhibitors (ICI) has substantially improved clinical outcomes in patients with advanced melanoma.^1 2^ After failure of first-line ICI treatment with anti-PD-1 antibodies, such as nivolumab or pembrolizumab, in patients with unresectable stage IIIC–IV cutaneous melanoma, second-line treatment with ipilimumab (anti-CTLA-4 antibody) monotherapy or ipilimumab/nivolumab combined has shown modest objective response rates up to 13% and 31%, respectively.^3 4^ For patients harboring a BRAF mutation (approximately 50% of melanomas), second-line treatment with BRAF/MEK inhibitors has shown better objective response rates of 22%–57%, but this clinical benefit is often short-lived.^5 6^ As limited clinical benefit is observed for second-line treatment with the currently available immuno and targeted therapies, there is a clear unmet medical need for novel treatment modalities for patients with unresectable stage IIIC–IV melanoma after first-line treatment failure.

Adoptive cell therapy with ex vivo-expanded tumor-infiltrating lymphocytes (TIL) from autologous melanoma tumors has been one of the first cell therapies developed with public funding by hospitals reaching advanced development milestones. Over the past years, multiple non-randomized single institution phase 2 clinical trials have been conducted in the USA, Israel and Europe, representing TIL as a safe and effective strategy to treat patients with metastatic melanoma.^7–12^ Recently, we showed statistically significant and clinically relevant improved progression-free survival (PFS) in patients with unresectable stage IIIC–IV melanoma after failed first-line or second-line treatment with tumor-infiltrating lymphocytes (TIL-NKI/CCIT) compared with ipilimumab, in a multicenter, open-label, randomized phase 3 clinical trial.^13^

Ex vivo-expanded TIL-NKI/CCIT are regulated as advanced therapy medicinal product (ATMP).^14^ As a consequence, the product needs to comply with stringent medicinal product quality, safety and efficacy standards. Therefore, substantial upfront public investments were needed, for example a fully equipped ATMP production facility with Good Manufacturing Practice-manufacturing license, skilled technical staff and controlled (hospital) logistics. To assess (financial) challenges, clinical implementation scenarios and an early cost-utility analysis (CUA) were conducted.^15–17^ The early-CUA estimated that TIL-NKI/CCIT was expected to yield more quality-adjusted life years (QALYs) at lower costs, based on early phase 2 clinical trial data and expert opinion. However, due to high evidentiary and clinical uncertainty, the need was expressed to reassess cost-effectiveness when confirmative clinical and cost data were available.

Therefore, the objective was to conduct a CUA based on a multicenter, open-label, randomized phase 3 clinical trial, calculating the incremental cost-effectiveness ratio (ICER) of treatment with TIL-NKI/CCIT compared with standard of care with ipilimumab in patients with unresectable stage IIIC–IV melanoma after failed first-line or second-line treatment. The phase 3 trial with TIL-NKI/CCIT was conducted as part of a coverage with evidence development (CED) program in the Netherlands in collaboration with Denmark.^9 18^

## Methods

### Study design

A prospective CUA was conducted assessing life years (LYs), QALYs and costs for patients with unresectable stage IIIC–IV melanoma after failed of first-line or second-line treatment comparing TIL-NKI/CCIT to standard of care treatment with ipilimumab. The CUA was based on the multicenter, open-label, randomized phase 3 clinical trial (TIL-NKI/CCIT trial: NCT02278887) conducted at the Netherlands Cancer Institute (NKI), the Netherlands, and the National Center for Cancer Immune Therapy (CCIT-DK), Denmark.^13^ A modified societal perspective was used with a lifetime horizon. The Dutch setting is presented as the base case, the Danish setting was included in a scenario analysis. Differences between the scenarios were country-specific costs. Methods and results adhere to the Consolidated Health Economic Evaluation Reporting Standards guidelines for cost-effectiveness analysis.

### Model description

**Figure 1:**
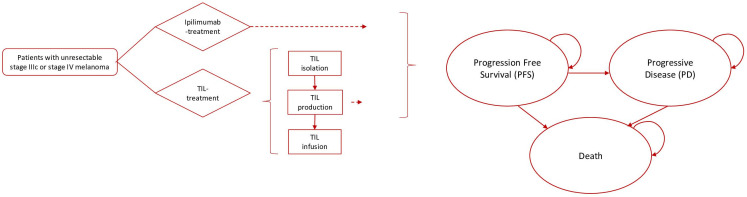
Model structure. Structure of the decision tree presented as a flow-diagram of treatment with ex vivo-expanded tumor infiltrating lymphocytes from autologous melanoma tumor (TIL-NKI/CCIT) and ipilimimab-arm combined with the Markov decision-model with three mutually exclusive health states: progression-free survival (PFS), progressive disease (PD) and the absorbing state death.

Treatment sequence was simulated by means of a Markov model. The Markov model included three mutually exclusive health states: PFS, progressive disease (PD) and death (all causes), see figure 1. In both treatment arms, all patients started in the PFS state. At the start of each cycle, patients could remain in the PFS state, could progress to PD or could progress to the absorbing death state. When progressed to PD, patients could remain in this state or progress to death. Cycle length was 3 months. Each cycle was associated with a health-state specific cost and utility, which accumulated over time. Base case model input parameters are reported in table 1, and a more detailed overview of input parameters is included in online supplemental table S1. PFS and PD probabilities over time per treatment arm are shown in online supplemental figure S1.

10.1136/jitc-2023-008372.supp1 Supplementary data

**Table 1:** Base case input parameters in Markov decision model for the Netherlands

**Costs tumor infiltrating lymphocytes (TIL-NKI/CCIT)**					
**Healthcare cost (progression free survival)**			**Base case**	**Unit**	**Source**
Screening			€ 3 822	Per patient	13 44
Physical examination			€ 2 917	Per patient	13 44
Lab tests			€ 607	Per patient	13 44
Consultations			€ 298	Per patient	13 44
TIL-NKI/CCIT isolation			€ 2,043	Per patient	13 44
Surgery			€ 1 583	Per patient	13 44
Hospital admission			€ 420	Per patient	13 44
Consultations			€ 40	Per patient	13 44
TIL-NKI/CCIT production			€ 67 547	Per product	13 44
Hospital admission and follow-up			€ 44 528	Per patient	13 44
Hospital admission			€ 20 706	Per patient	13 44
Medications			€ 12 190	Per patient	13 44
Laboratory tests			€ 5 004	Per patient	13 44
Blood products			€ 1 926	Per patient	13 44
Consultations			€ 205	Per patient	13 44
Specialized nurse Others (eg, ECG, CT, chest X-ray, supportive care)			€ 2 429 € 2 069	Per patient Per patient	13 44
Total costs TIL-NKI/CCIT treatment			€ 117 940	Per patient	
Healthcare costs (progressive disease)*			**Base case**	**Unit**	**Source**
Ipilimumab monotherapy			€ 66 388	0.20	13 44
BRAF/MEK inhibitor			€ 101 224	0.20	13 44
Ipilimumab/nivolumab combination therapy			€ 72 514	0.11	13 44
Pembrolizumab			€ 54 571	0.01	13 44
No treatment			€ 0	0.43	13 44
Other (temozolomide, ipilimumab/pembrolizumab)			€ 96 448	0.05	13 44
**Death**			**Base case**	**Unit**	**Source**
Costs associated with (3 months prior to) death			€ 1 516	Per patient	13 44
**Societal costs**			**Base case**	**Unit**	**Source**
Direct patient costs (medication, homecare, travel)			€ 227	First cycle	13 44
Direct patient costs (medication, homecare, travel)			€ 82	>First cycle	13 44
Direct patient costs (copay)			€ 385	Per year	13 44
Informal care			€ 710	First cycle	13 38 44
Informal care			€ 99	>First cycle	13 38 44
Productivity loss			€ 3 539	First cycle	13 38 44
Productivity loss			€ 75	>First cycle	13 38 44
**Costs ipilimumab**					
**Healthcare costs (progression-free survival)**			**Base case**	**Unit**	**Source**
Screening			€ 2 507	Per patient	13 44
Physical examination and lab tests			€ 2 507	Per patient	13 44
Ipilimumab treatment			€ 75 316	Per patient	13 44
Hospital admission			€ 3 200	Per patient	13 44
Ipilimumab, including supportive medicines			€ 66 388	Per patient	13 44
Lab tests			€ 2 103	Per patient	13 44
Blood products			€ 105	Per patient	13 44
Consultations			€ 648	Per patient	13 44
Others (eg, ECG, CT, chest X-ray, supportive care)			€ 2 872	Per patient	13 44
Total costs ipilimumab treatment			€ 77 823	Per patient	
Healthcare costs (progressive disease)*			**Base case**	**Unit**	**Source**
Ipilimumab rechallenge			€ 66 388	0.02	13 44
BRAF/MEK inhibitor			€ 101 224	0.29	13 44
Pembrolizumab			€ 54 571	0.10	13 44
No treatment/other trial			€ 0	0.57	13 44
Other (dacarbazine, temozolomide)			€ 6 814	0.02	13 44
**Death**			**Base case**	**Unit**	**Source**
Costs associated with (3 months prior to) death			€ 1 516	Per patient	13 44
**Societal costs**			**Base case**	**Unit**	**Source**
Direct patient costs (medication, homecare, travel)			€ 210	First cycle	13 44
Direct patient costs (medication, homecare, travel)			€ 27	>First cycle	13 44
Direct patient costs (copay)			€ 385	Per year	13 44
Informal care			€ 916	First cycle	13 38 44
Informal care			€ 99	>First cycle	13 38 44
Productivity loss			€ 3 539	First cycle	13 38 44
Productivity loss			€ 75	>First cycle	13 38 44
**Survival (probabilities)**					
	**TIL**		**Ipilimumab**		
	**Modeled** **PFS†**	**Modeled** **OS†**	**Modeled PFS†**	**Modeled** **OS†**	**Source**
Baseline	1.000	1.000	1.000	1.000	13 44
Month 3	0.792	0.942	0.635	0.936	13 44
Month 6	0.612	0.871	0.269	0.847	13 44
Month 9	0.485	0.801	0.129	0.759	13 44
Month 12†	0.395	0.735	0.072	0.679	13 44
**Utilities**					
**Stable disease**			**Base case**	**Unit**	**Source**
TIL-NKI/CCIT: baseline			0.874	Per cycle	13 24 45
TIL-NKI/CCIT: month 3			0.879	Per cycle	13 24 45
TIL-NKI/CCIT: month 6			0.885	Per cycle	13 24 45
TIL-NKI/CCIT: month 9			0.881	Per cycle	13 24 45
TIL-NKI/CCIT: month 12†			0.887	Per cycle	13 24 45
Ipilimumab: baseline			0.838	Per cycle	13 24 45
Ipilimumab: month 3			0.840	Per cycle	13 24 45
Ipilimumab: month 6			0.841	Per cycle	13 24 45
Ipilimumab: month 9			0.849	Per cycle	13 24 45
Ipilimumab: month 12‡			0.828	Per cycle	13 24 45
**Progressive disease**			**Base case**	**Unit**	**Source**
Ipilimumab (rechallenge)			0.764	Per cycle	32
Ipilimumab/nivolumab combination therapy			0.695	Per cycle	46
BRAF/MEK inhibitor			0.844	Per cycle	47
Pembrolizumab			0.707	Per cycle	48
Temozolomide			0.730	Per cycle	49
Dacarbazine			0.791	Per cycle	50
No treatment after TIL-NKI/CCIT			0.832	Per cycle	13
No treatment after ipilimumab			0.764	Per cycle	13
Death (applied to 3 months prior to death)			0.665	Per cycle	40

*Healthcare costs progressive disease: costs for nivolumab and ipilimumab were based on the schedule 1 mg/kg nivolumab+3 mg/kg ipilimumab every 3 weeks for four cycles, followed by 240 mg/2 weeks per model cycle until progression or death. Costs for BRAF/MEK were based on the regimen dabrafenib (150 mg/2dd)/trametinib (2 mg/1dd). Pembrolizumab was based on 200 mg intravenous every 3 weeks cycle until progression or death. Temozolomide regimen was based on 150 mg/m^2^ two times a day for 7 days for four cycles and dacarbazine 850 mg/m^2^ for three cycles. ±PFS and OS estimates are derived from the TIL-NKI/CCIT-study and modeled to fit and extrapolated beyond the trial time horizon using a log-logistic distribution.

†PFS and OS estimates are derived from the TIL-NKI/CCIT-study and modeled to fit and extrapolated beyond the trial time horizon using a log-logistic distribution.

‡PFS, OS and utility values are beyond 12 months and reported in more detail elsewhere.^13^ A formal data request can be directed to the NKI/AvL. For terms and procedure, we refer to the data sharing agreement in the initial publication.^13^

BRAF/MEK, v-Raf murine sarcoma viral oncogene homolog B1/mitogen activated protein kinase; OS, overall survival; PFS, progression-free survival; TIL-NKI/CCIT, ex vivo-expanded tumor infiltrating lymphocytes from autologous melanoma tumor.

### Patients

After eligibility screening for the TIL-NKI/CCIT-trial, 168 patients were randomly assigned to receive either TIL-NKI/CCIT treatment (84 patients) or standard ipilimumab treatment (84 patients). Of 168 patients, 132 were treated in the Netherlands and 36 in Denmark, with an even distribution between treatments arms. Baseline characteristics were well-balanced in both study groups.^13^ The primary trial endpoint was PFS, as defined by Response Evaluation Criteria In Solid Tumors (RECIST), version 1.1.

### Intervention and comparator

The intervention of interest was TIL-NKI/CCIT cell therapy. After randomization to the TIL-NKI/CCIT treatment arm, patients underwent surgical resection of a melanoma metastasis for TIL-NKI/CCIT production according to the TIL-NKI/CCIT-treatment protocol (figure 1). This was followed by TIL-NKI/CCIT infusion, preceded by non-myeloablative lymphodepleting chemotherapy.^13^ TIL-NKI/CCIT infusion consisted of the single intravenous adoptive transfer of at least 5×10^9^ autologous TILs, followed by administration of high-dose bolus interleukin 2 (600.000 IU/kg/dose every 8 hours intravenous with a maximum of 15 doses). Supportive care comprised thrombocyte and erythrocyte transfusions until spontaneous hematopoietic recovery. The trial showed that after disease progression on TIL-NKI/CCIT therapy, 20% of patients switched to ipilimumab monotherapy, 20% to BRAF/MEK combination therapy, 11% to ipilimumab/nivolumab combination therapy, 1% to pembrolizumab, 5% to other treatments (25% temozolomide, 75% ipilimumab/pembrolizumab) and the remaining 43% received no further treatment (table 1 and online supplemental table S1).

TIL-NKI/CCIT treatment was compared with ipilimumab, which was standard of care for this population at time of trial initiation. Ipilimumab was administered according to the standard regimen of 3 mg/kg intravenous once every 3 weeks, for maximum of four cycles.^19–21^ After progression on ipilimumab treatment, 29% of patients continued on BRAF/MEK combination therapy, 10% on pembrolizumab, 2% on ipilimumab (rechallenge), 2% on other treatments (50% dacarbazine, 50% temozolomide) and the remaining 57% of patients continued to participate in another clinical trial (20%) or received no further treatment (37%), see table 1 and online supplemental table S1.

### Health effects

#### Survival

Health state transition probabilities were informed by PFS and overall survival (OS) data from the TIL-NKI/CCIT trial.^13^ The cut-off for clinical data collection was June 9, 2022. Of the survival curves, log-cumulative hazard plots were compared for initial model selection and extrapolated beyond trial duration by fitting individual parametric survival curves. Best fit was assessed according to the Akaike Information Criterion, Bayesian Information Criterion and visual (expert) inspection.^22 23^ This resulted in the selection of the log-logistic, see online supplemental table S2. Online supplemental fi gure S1 includes PFS and PD probabilities over time and best fitted parametric survival curves. The model was cut-off if patients reached the age of 100 years or 99.9% of patients were deceased.

#### Health state utilities

For each treatment arm, QALYs were calculated based on utilities. A utility is a standardized score between 0 and 1, with 0 reflecting death and 1 perfect health, measured via the EuroQol 5D-3L (EQ-5D) questionnaire. When utility is multiplied by length of survival, it yields a QALY. To derive utilities from the EQ-5D questionnaire, country-specific Dutch and Danish EQ-5D tariffs were applied to the appropriate cohorts.^24 25^ After disease progression, QALY measurements were discontinued as per study protocol. For patients continuing to a next line of treatment, a treatment-specific utility was extracted from literature (table 1 and online supplemental table S1).^26–29^

### Resource use and costs

#### Treatment costs

For the Dutch study population, the TIL-NKI/CCIT products were developed and manufactured at the NKI and Sanquin Bloodbank, therefore no formal list price was available. The costs per TIL-NKI/CCIT product were therefore calculated via the cell therapy manufacturing cost framework using activity-based costing.^30 31^ A detailed cost calculation was conducted in each center, more detail is included in online supplemental methods.

Ipilimumab treatment costs comprised drug costs, patient hospital admission for treatment and supportive medication (ie, infliximab for adverse events). Base cases were calculated by matching trial informed population mean dosage, patient weight and admission frequency to Dutch 2021 drug list tariffs, see table 1 and online supplemental table S1.

#### Healthcare resources

Healthcare utilization included physical examinations, hospital admissions, laboratory tests, blood products, imaging and (surgical) interventions (table 1 and online supplemental table S1). Occurrence and frequency of consumed resources was informed by the TIL-NKI/CCIT-trial and extracted from hospital records. In the base case, resource use was multiplied with Dutch 2021 unit costs in Euros (€), in adherence with the Dutch guideline on costing research in healthcare. As per trial protocol, post-treatment follow-up was modeled concurrently with clinical and health-related quality of life questionnaire follow-up. These follow-ups were assumed to stop after 5 years, as ongoing medical insight reports that patients without signs of PD after 5 years are considered cured.^32^

#### Non-healthcare related costs

In adherence to the modified societal perspective, non-hospital related healthcare costs, out-of-pocket expenses and treatment-related travel costs were also included. In the first health-related quality of life follow-up questionnaire, patients were asked once to report costs related to travel, medication and homecare. It was assumed these were representative of the preceding years. In addition, costs of productivity loss (absenteeism) were included for patients who reported employment (42%, average 0.52 fte) for the 2021 friction period (78.9 days).^33 34^ In addition, productivity loss and travel expenses were included for family and friends, assuming that in 50% of hospital visits a patient was accompanied by someone who had to take half a day off.

### Statistical analysis

The model was constructed in Microsoft Excel, 2010 (Microsoft, Redmond, WA). Incremental benefits and costs between treatment arms were expressed using the ICER and were calculated based on the intention-to-treat analysis of the TIL-NKI/CCIT-trial.^13^ The ICER captures the incremental costs per full QALY gained of an intervention compared with another: ICER=(Costs_intervention_–Costs_StandardOfCare_)/(QALY_intervention_–QALY_StandardOfCare_).

Further statistical analyses were performed using R statistical computing V.4.0.3 (R Foundation for Statistical Computing, Vienna, Austria). QALYs over time were modeled via generalized estimating equations.^35 36^ Missing items from the EQ-5D were imputed according to the EQ-5D scoring guidelines by using R multivariate imputation via chained equation package, using baseline characteristics, treatment outcome, EQ-5D-questionnaires and costs as predictors.^37^ Dutch discount rates were applied in the base case; 4.0% on costs and 1.5% on benefits (LYs and QALYs).^38^ Discounting was applied to convert future costs and effects to their present value.^39^

### Sensitivity analyses

Uncertainty around parameter estimates were explored via deterministic (DSA) and probabilistic sensitivity analyses (PSA). The DSA explores the impact of individual parameters by alternately varying input values between pre-set minimum and maximum values and can be considered parameter-specific best-case and worst-case scenarios. Minimum and maximum values were informed by 95% CIs or variance of the mean by ±20% (table 1 and online supplemental table S1).

A PSA provides a more comprehensive uncertainty estimate by simultaneously sampling all parameters. This was done by sampling 10,000 iterations of all model input parameters according to their individual appropriate minimum, maximum and distributions. In addition, here the PSA was presented with a relative density plot to better visualize uncertainty and clustering of ICER-estimates. With the PSA output, the probability of a treatment being cost-effective for a given willingness-to-pay (WTP) threshold was estimated, which is presented as a cost-effectiveness acceptability curve (CEAC). The Dutch informal WTP of €80,000/incremental QALY was applied.^40^

### Scenario analysis

In addition to the Dutch base case analysis, a scenario analysis was conducted in which costs for the Danish setting were included, see online supplemental table S3. The survival and quality of life input parameters in the scenario were the same as in the Dutch base case setting (table 1 and online supplemental table S1). Costs were estimated by multiplying mean population used drug and healthcare utilization with Danish 2021 tariffs and converted from Danish kroner (DKK) to Euros (€). Also, country specific discount rates of 3.5% for both costs and (QA)LYs were applied in line with methods of the Danish Medicines Council, and an adjusted friction period (75.4 days) was applied to estimate productivity loss.^33 34 38 41^ In Denmark, no WTP-threshold has been reported. Therefore, The WHO’s Choosing Interventions that are Cost-Effective recommendation was adopted, which states that an intervention with a cost/QALY of less than the national annual gross domestic product (GDP) per capita, is considered cost-effective. This translates to an informal threshold of €50,000/incremental QALY based on the Danish GDP per capital as of December 2021.

## Results

### Benefits and costs of treatments

Higher total LYs, QALYs and lower total costs for the TIL-NKI/CCIT treatment compared with treatment with ipilimumab were observed in both the Dutch base case and Danish scenario analysis. Treatment with TIL-NKI/CCIT resulted in mean undiscounted LYs of 4.47 (95% credibility interval (CrI) 3.88–5.29) and QALYs of 3.52 (95% CrI 3.30–4.59) compared with 3.33 (95% CrI 2.88–4.00) LY and 2.46 (95% CrI 1.47–3.41) QALYs for patients treated with ipilimumab (table 2 and online supplemental table S4).

**Table 2:** Undiscounted and discounted base case life years, quality adjusted life years, costs and incremental cost-effectiveness ratios of TIL-NKI/CCIT-treatment compared with Ipilimumab in the Netherlands

	Undiscounted			Discounted*		
	TIL-NKI/CCIT	Ipilimumab	Incremental	TIL-NKI/CCIT	Ipilimumab	Incremental
5 years	Mean					
Life years	2.46	2.11	0.35	2.40	2.06	0.34
QALYs	1.94	1.53	0.41	1.89	1.49	0.40
Costs	€ 224,502	€ 283,100	€ −58,599	€ 214,089	€ 266,455	€ −52,366
ICER	Dominant			Dominant		
10 years						
Life years	3.38	2.72	0.66	3.23	2.61	0.62
QALYs	2.66	1.99	0.67	2.55	1.91	0.64
Costs	€ 279,683	€ 358,242	€ −78,559	€ 255,931	€ 323,591	€ −67,660
ICER	Dominant			Dominant		
Lifetime						
Total LYs	4.47	3.33	1.14	4.09	3.10	0.99
Total QALYs	3.52	2.46	1.06	3.22	2.28	0.94
Total costs	€ 347,168	€ 433,634	€ −86,467	€ 292,369	€ 365,068	€ −72,699
ICER	Dominant			Dominant		

Calculated ICER=(Costs_intervention_–Costs_StandardOfCare_)/(QALY_intervention_–QALY_StandardOfCare_).

*Costs are discounted with 4% per year and effects (life years and QALYs) with 1.5% per year in line with Dutch guidelines for economic evaluations. Discounting is applied to adjust future costs and effects to their present value.

ICER, incremental cost-effectiveness ratio; QALYs, quality-adjusted life years; TIL-NKI/CCIT, ex vivo-expanded tumor infiltrating lymphocytes from autologous melanoma tumor.

In the Netherlands, mean lifetime undiscounted societal costs per patient were €347,168 (95% CrI €269,889–447,100) for TIL-NKI/CCIT compared with €433,634 (95% CrI €329,255–571,253) for ipilimumab, and in the Danish scenario €337,309 (95% CrI €264,324–438,132) versus €436,135 (95% CrI €331,304–€572,222), respectively (table 2 and online supplemental table S4). Adjusted benefits and costs with country-specific discount rates are presented in table 2 and online supplemental table S4.

Although initial costs of TIL-NKI/CCIT production and treatment-related healthcare costs were higher in the base case and scenario than ipilimumab costs, the cost savings seem to be driven by longer observed and modeled PFS in the TIL-NKI/CCIT cohort. Prolonged PFS consequently delayed or prevented the need for additional care and switching to (more costly) next-line oncological treatments.

### Deterministic and probabilistic sensitivity analyses

**Figure 2:**
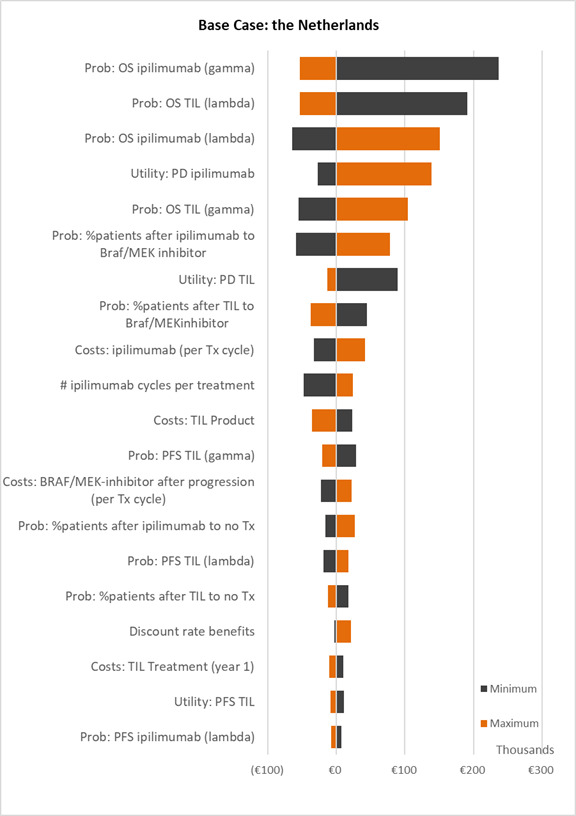
Deterministic (univariate) sensitivity analysis. Results of the deterministic (univariate) sensitivity analysis visualized in a tornado diagram. The diagram shows the impact of discounted individual parameters on the incremental cost-effectiveness ratio by alternately varying input values one by one between pre-set minimum and maximum values (see table 1). BRAF/MEK, V-Raf murine sarcoma viral oncogene homolog B1/mitogen activated protein kinase; ipi, ipilimumab; OS, overall survival; PFS, progression-free survival; Prob, probability; QALY, quality-adjusted life year; TIL-NKI/CCIT, ex vivo-expanded tumor infiltrating lymphocytes from autologous melanoma tumor; Tx, treatment; #, number.

The result of the base case DSA is shown in the tornado diagram (figure 2). The parameters with the biggest impact on the ICER were survival probabilities, quality of life in PD and next-line treatment cost in PD. The DSA for the Danish scenario is included in online supplemental figure S2 and shows similar results.

**Figure 3:**
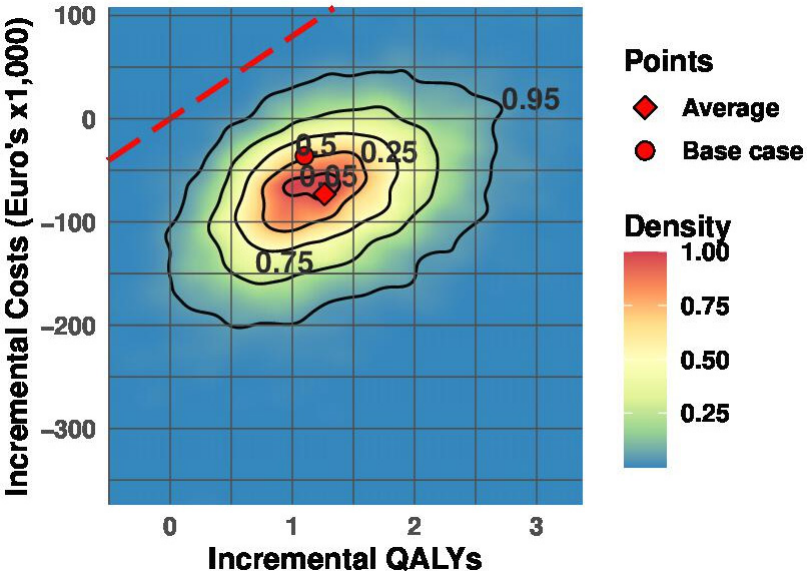
Probabilistic sensitivity analysis. Results of the probabilistic sensitivity analysis (PSA) visualized in cost-effectiveness plane. The PSA shows uncertainty of estimated discounted base case incremental cost-effectiveness ratio over a lifetime horizon by simultaneously sampling uncertainty across all parameters by 10,000 iterations. All model input parameters are sampled randomly, according to their individual appropriate distributions between pre-set minimum and maximum values (table 1). QALYs, quality-adjusted life years; WTP, willingness to pay.

The probability of TIL-NKI/CCIT being cost-effective compared with ipilimumab, at a WTP-threshold of €80,000 per QALY, was >99% in the Dutch base case, as can be seen in the CEAC (online supplemental figure S3). The PSA results are visualized in figure 3. This plot shows that almost all simulated ICER estimates are located in the south-east quadrant of the cost-effectiveness plane, and no ICER-estimates are located above the WTP-threshold. The CEAC for the Danish setting is shown in online supplemental figure S4 and shows that, in line with the PSA in online supplemental figure S5, at a WTP of €50,000, the chance of TIL-NKI/CCIT being cost-effective in comparison to ipilimumab, is 99%. Despite the uncertainty quantified in the input parameters, the likelihood of treatment with TIL-NKI-CCIT compared with ipilimumab being cost-effective is ≥99% in both the Danish and Dutch situation. Moreso, in the base case as well as in a large proportion of estimates it is even cost-saving, while providing additional health benefits as treatment with more expensive oncological agents is avoided or postponed.

## Discussion

Based on the multicenter, randomized phase 3 trial, treatment with TIL-NKI/CCIT for patients with unresectable stage IIIC–IV melanoma, with the majority of patients with failed first-line treatment, is accompanied by a substantial gain in QALYs and by cost-savings in both a Dutch and Danish setting compared with treatment with standard of care ipilimumab.^13^ This indicates that TIL-NKI/CCIT therapy is cost-effective compared with standard ICI immunotherapy with ipilimumab for this patient population. In addition, to the best of our knowledge, this is the first cost-effectiveness analysis of a novel cell therapy developed by public funds, based on data from a randomized clinical phase 3 trial.

The CUA in this study was performed as part of a CED program.^18^ Within this program, a comprehensive Health Technology Assessment (HTA) was performed to identify challenges and to support timely access to the promising innovation. This HTA included a qualitative study assessing aspects that play a role in the implementation of cell therapies, where we found that public financing and a multidisciplinary approach was conditional.^19^ (30) Furthermore, an early CUA was performed, followed by a scenario study where several future development scenarios were incorporated.^16 17^ The results from the early CUA, which estimated that TIL-NKI/CCIT therapy could be cost-effective compared with treatment with ipilimumab, hold in the current trial-based CUA. Herewith, we demonstrated that conducting early economic evaluations—including scenarios—was informative and can support the design of product development strategies.

To estimate the costs per TIL-NKI/CCIT product, activity-based costing was applied. Production costs between small facilities differ greatly and are highly facility dependent.^31^ The costs per TIL-NKI/CCIT product presented here reflect true production costs during the clinical trial without marketing costs, (profit) margins, costs for obtaining and maintaining marketing authorization or early investments and will certainly be an underestimation compared with a more commercial setting.^31^ This means that if changes occur in organization or demand, it directly impacts the here presented production cost estimates. A TIL-NKI/CCIT adoption scenario analysis based on expert opinion, conducted as part of the CED, assumed commercial costs of TIL-NKI/CCIT would be at least three times higher.^17^ According to DSA results, if this assumption is applied, TIL-NKI/CCIT still remains the most cost-effective option compared with ipilimumab. However, in the context of increased interest in public funded cell therapy development, further research on efficiency, organization, upscaling of manufacturing and costing is needed.

The trial-based CUA design of this study provides a direct comparison between treatment arms and allows to take into account real-world (cost) data. This affected our analysis as guidelines describe four cycles of ipilimumab treatment in this population.^20^ Our data showed that patients received on average three cycles of ipilimumab due to ipilimumab-induced toxicity or rapid PD.^13^ This observation is in line with previous reports of real-world use of ipilimumab.^42^ Given the high costs of ipilimumab, the estimated cost-saving in this study might be an underestimation in populations that more often receive four cycles of ipilimumab. Although a trial-based analysis has numerous advantages, a limitation is its limited time horizon. To extrapolate trial data beyond trial duration to a lifetime horizon, assumptions were made regarding future follow-up activities. In addition, the optimal time to determine OS of both treatment modalities was not yet reached.^13^ Although a positive trend is visible in OS for TIL-NKI/CCIT therapy compared with ipilimumab, this difference did not (yet) reach statistical significance, despite profound differences in PFS.^13^ Consequently, the beneficial effect on survival on TIL-NKI/CCIT treatment reflected in the (QA)LYs might currently be underestimated. Furthermore, information on third and following treatment lines were available on a patient level, but associated costs and utilities were literature-derived due to the trial design. Also, indirect costs were not collected in line with current HTA-guidelines (the trial was designed under the 2010 Dutch guideline for costing studies), therefore assumptions were made to adhere to the societal perspective. Additionally, as often seen with clinical trials, the standard of care for patients with melanoma has moved from ipilimumab monotherapy to ipilimumab/nivolumab combination therapy for a specific patient population during the course of the trial.^19^ As there is no head-to-head comparisons yet available of ipilimumab/nivolumab versus TIL-NKI/CCIT, the cost-effectiveness of this comparator was not included in this study. As the price of ipilimumab/nivolumab is substantial, a large difference in survival is necessary to change the conclusion of this study. Whether our hypothesis stands will need to be clinically confirmed. Research is ongoing and results of a head-to-head trial comparing ipilimumab and ipilimumab/nivolumab in a similar population are expected in 2024/2025.^43^

### Clinical interpretation

Although the mean costs of TIL-NKI/CCIT treatment (including TIL-NKI/CCIT production and hospital admission) itself were higher compared with ipilimumab in patients with metastatic melanoma, the considerable increase in observed and modeled survival, delayed or forgone need for additional care, and (more costly) next-line treatment led to overall cost savings. This demonstrates the need to assess clinical and economic impact of new therapies not just based on its initial costs, but also on their impact on the healthcare and care pathways as a whole.

Even though advancements of metastatic melanoma treatment move towards ipilimumab/nivolumab combination therapy for a part of the population, the results of the phase 3 trials and this cost-effectiveness analysis have demonstrated impact on treatment guidelines in the Netherlands and Denmark. However, large scale implementation of TIL-NKI/CCIT treatment may still be associated with production and logistical challenges due to its personalized nature. Therefore, continued efforts are needed to address these challenges. This is emphasized, as TIL-NKI/CCIT therapy is one of the first cell therapies developed by two hospitals using public financing and is moving towards European market access.

To conclude, TIL-NKI/CCIT treatment for patients with unresectable stage IIIC–IV cutaneous melanoma failed first-line or second-line treatment showed gained QALYs against less costs, both in a Dutch and Danish setting in comparison to treatment with standard ipilimumab. This supported reimbursement of TIL-NKI/CCIT as well as impacted treatment guidelines in this patient population. In addition, development of cell therapies by research institutes and hospitals funded by public money show realistic promise to further explore effective personalized treatment while warranting the economic sustainability of healthcare systems.

## Data Availability

Data are available upon reasonable request.
