# Supplementary material for: Cost-effectiveness of treating advanced melanoma with tumor-infiltrating lymphocytes based on an international randomized phase 3 clinical trial
Source: J Immunother Cancer. 2024 Mar 26;12(3):e008372. doi: 10.1136/jitc-2023-008372 (PMC10966812; doi:10.1136/jitc-2023-008372)
Supplement: Supplementary data [file jitc-2023-008372supp001.pdf]

Cost-effectiveness of treating advanced melanoma with tumor-infiltrating lymphocytes based on an international randomized phase 3 clinical trial

Authors: Renske M.T. ten Ham[a,b], Maartje W. Rohaan[c], Inge Jedema[d], Rob Kessels[e], Wim Stegeman[e], Walter Scheepmaker[f], Bastiaan Nuijen[g], Cynthia Nijenhuis[h], Melanie Lindenberg[a], Troels Holz Borch[i], Tine Monberg[i], Marco Donia[i], Inge Marie Svane[i], Wim van Harten[a,j], John Haanen[c,k], Valesca P. Retèl[a,l]

Affiliations: [a]Netherlands Cancer Institute, Division of Psychosocial Research and Epidemiology, Amsterdam, The Netherlands, [b]University Medical Centre Utrecht, Julius Centre, Department of Epidemiology & Health Economics, Utrecht, The Netherlands, [c]Netherlands Cancer Institute, Division of Medical Oncology, Amsterdam, The Netherlands, [d]Netherlands Cancer Institute, Division of Molecular Oncology and Immunology, Amsterdam, The Netherlands, [e]Netherlands Cancer Institute, Department of Biometrics, Amsterdam, The Netherlands, [f]Netherlands Cancer Institute, Financial department, Amsterdam, The Netherlands, [g]Netherlands Cancer Institute, Division of Pharmacy and Pharmacology, Amsterdam, The Netherlands, [h]Netherlands Cancer Institute, Biotherapeutics Unit, Amsterdam, The Netherlands, [i]National Center for Cancer Immune Therapy, Department of Oncology, Copenhagen University Hospital, Herlev, Denmark, [j]Department of Health Technology and Services Research, University of Twente, Enschede, The Netherlands, [k]Leiden University Medical Center, Department of Clinical Oncology, Leiden, The Netherlands [l]Erasmus University Rotterdam, Erasmus School of Health Policy and Management, Rotterdam, The Netherlands

Corresponding author: Prof. Dr. Valesca P. Retèl, The Netherlands Cancer Institute, Department of Psychosocial Research and Epidemiology, Plesmanlaan 121, 1066CX Amsterdam, The Netherlands, Phone number: 0031 205126197. Email address: v.retel@nki.nl

Article type: Original Research

Running head: Cost-effectiveness of tumor-infiltrating lymphocytes for advanced melanoma

Supplemental Materials

Table S1: Detailed base case input parameters in Markov decision model for the Netherlands.

**Costs Tumor infiltrating lymphocytes (TIL-NKI/CCIT)**						
Healthcare cost (progression free survival)		*Base Case*	*Unit*	*Min*	*Max*	*Source*
	Screening	€ 3,822	Per patient	€ 3,058	€ 4,586	11, 43
	Physical examination	€ 2,917	Per patient	€ 2,334	€ 3,500	11, 43
	Lab tests	€ 607	Per patient	€ 486	€ 728	11, 43
	Consultations	€ 298	Per patient	€ 238	€ 357	11, 43
	TIL-NKI/CCIT isolation	€ 2,043	Per patient	€ 1,634	€ 2,452	11, 43
	Surgery	€ 1,583	Per patient	€ 1,266	€ 1,899	11, 43
	Hospital admission	€ 420	Per patient	€ 336	€ 504	11, 43
	Consultations	€ 40	Per patient	€ 32	€ 48	11, 43
	TIL-NKI/CCIT production	€ 67,547	Per product	€ 45,031	€ 101,320	11, 43
	Hospital admission and follow-up	€ 44,528	Per patient	€ 35,622	€ 53,433	11, 43
	Hospital admission	€ 20,706	Per patient	€ 16,565	€ 24,847	11, 43
	Medications	€ 12,190	Per patient	€ 9,752	€ 14,628	11, 43
	Laboratory tests	€ 5,004	Per patient	€ 4,003	€ 6,004	11, 43
	Blood products	€ 1,926	Per patient	€ 1,541	€ 2,311	11, 43
	Consultations	€ 205	Per patient	€ 164	€ 246	11, 43
	Specialized nurse	€ 2,429	Per patient	€ 1,943	€ 2,914	11, 43
	Others (e.g., ECG, CT, chest X-ray, supportive care)	€ 2,069	Per patient	€ 1,655	€ 2,483	11, 43
*Health care costs (progressive disease)**		*Base Case*	*Unit*	*Min*	*Max*	*Source*
	Ipilimumab monotherapy	€ 66,388	0.20	€ 35,451	€ 106,156	11, 43
	BRAF/MEK inhibitor	€ 101,224	0.20	€ 80,980	€ 121,469	11, 43
	Ipilimumab/nivolumab combination therapy	€ 72,514	0.11	€ 58,011	€ 87,017	11, 43
	Pembrolizumab	€ 54,571	0.01	€ 43,656	€ 65,485	11, 43
	No treatment	€ 0	0.43	€ 0	€ 5,000	11, 43
	Other (temozolomide, ipilimumab/pembrolizumab)	€ 96,448	0.05	€ 77,159	€ 115,738	11, 43
Death		*Base Case*	*Unit*	*Min*	*Max*	*Source*
	Costs associated with (3 months prior to) death	€ 1,516	Per patient	€ 1,213	€ 1,820	11, 43
Societal costs		*Base Case*	*Unit*	*Min*	*Max*	*Source*
	Direct patient costs (medication, homecare, travel)	€ 227	First cycle	€ 182	€ 273	11, 43
	Direct patient costs (medication, homecare, travel)	€ 82	>first cycle	€ 65	€ 98	11, 43
	Direct patient costs (co pay)	€ 385	Per year	€ 385	€ 985	11, 43
	Informal care	€ 710	First cycle	€ 568	€ 851	11,36,43
	Informal care	€ 99	>first cycle	€ 79	€ 118	11,36,43
	Productivity loss	€ 3,539	First cycle	€ 1,450	€ 7,338	11,36,43
	Productivity loss	€ 75	>first cycle	€ 60	€ 91	11,36,43
**Costs ipilimumab**						
Healthcare costs (progression-free survival)		*Base Case*	*Unit*	*Min*	*Max*	*Source*
	Screening	€ 2,507	Per patient	€ 2,005	€ 3,008	11, 43
	Physical examination and lab tests	€ 2,507	Per patient	€ 2,005	€ 3,008	11, 43
	Ipilimumab treatment	€ 75,316	Per patient	€ 42,593	€ 116,870	11, 43
	Hospital admission	€ 3,200	Per patient	€ 2,560	€ 3,841	11, 43
	Ipilimumab, including supportive medicines	€ 66,388	Per patient	€ 35,451	€ 106,156	11, 43
	Lab tests	€ 2,103	Per patient	€ 1,682	€ 2,524	11, 43
	Blood products	€ 105	Per patient	€ 84	€ 125	11, 43
	Consultations	€ 648	Per patient	€ 518	€ 777	11, 43
	Others (e.g., ECG, CT, chest X-ray, supportive care)	€ 2,872	Per patient	€ 2,298	€ 3,447	11, 43
*Health care costs (progressive disease)**		*Base Case*	*Unit*	*Min*	*Max*	*Source*

Ipilimumab rechallenge	€ 66,388	0.02	€ 35,451	€ 106,156	11, 43
BRAF/MEK inhibitor	€ 101,224	0.29	€ 80,980	€ 121,469	11, 43
Pembrolizumab	€ 54,571	0.10	€ 43,656	€ 65,485	11, 43
No treatment/other trial	€ 0	0.57	€ 0	€ 5,000	11, 43
Other (dacarbazine, temozolomide)	€ 6,814	0.02	€ 5,451	€ 8,176	11, 43
Death	*Base Case*	*Unit*	*Min*	*Max*	*Source*
Costs associated with (3 months prior to) death	€ 1,516	Per patient	€ 1,213	€ 1,820	11, 43
Societal costs	*Base Case*	*Unit*	*Min*	*Max*	*Source*
Direct patient costs (medication, homecare, travel)	€ 210	First cycle	€ 168	€ 252	11, 43
Direct patient costs (medication, homecare, travel)	€ 27	>first cycle	€ 21	€ 32	11, 43
Direct patient costs (co pay)	€ 385	Per year	€ 385	€ 985	11, 43
Informal care	€ 916	First cycle	€ 733	€ 1100	11,36,43
Informal care	€ 99	>first cycle	€ 79	€ 118	11,36,43
Productivity loss	€ 3,539	First cycle	€ 1,450	€ 7,338	11,36,43
Productivity loss	€ 75	>first cycle	€ 60	€ 91	11,36,43
**Survival**					
	TIL		ipilimumab		
	Modelled PFS±	Modelled OS±	Modelled PFS±	Modelled OS±	*Source*
baseline	1.000	1.000	1.000	1.000	11, 43
month 3	0.792	0.942	0.635	0.936	11, 43
month 6	0.612	0.871	0.269	0.847	11, 43
month 9	0.485	0.801	0.129	0.759	11, 43
month 12^	0.395	0.735	0.072	0.679	11, 43
**Utilities**					
Stable disease	*Base Case*	*Unit*	*Min*	*Max*	*Source*
TIL-NKI/CCIT: Baseline	0.874	Per cycle	0.870	0.878	11,44,45
TIL-NKI/CCIT: month 3	0.879	Per cycle	0.873	0.886	11,44,45
TIL-NKI/CCIT: month 6	0.885	Per cycle	0.879	0.892	11,44,45
TIL-NKI/CCIT: month 9	0.881	Per cycle	0.872	0.889	11,44,45
TIL-NKI/CCIT: month 12^	0.887	Per cycle	0.878	0.896	11,44,45
Ipilimumab: Baseline	0.838	Per cycle	0.835	0.842	11,44,45
Ipilimumab: month 3	0.840	Per cycle	0.833	0.843	11,44,45
Ipilimumab: month 6	0.841	Per cycle	0.825	0.847	11,44,45
Ipilimumab: month 9	0.849	Per cycle	0.822	0.864	11,44,45
Ipilimumab: month 12^	0.828	Per cycle	0.811	0.862	11,44,45
Progressive disease	*Base Case*	*Unit*	*Min*	*Max*	*Source*
Ipilimumab (rechallenge)	0.764	Per cycle	0.611	0.917	30
Ipilimumab/nivolumab combination therapy	0.695	Per cycle	0.556	0.834	46
BRAF/MEK inhibitor	0.844	Per cycle	0.820	0.867	47
Pembrolizumab	0.707	Per cycle	0.566	0.848	48
Temozolomide	0.730	Per cycle	0.584	0.876	49
Dacarbazine	0.791	Per cycle	0.633	0.949	50
No treatment after TIL-NKI/CCIT	0.832	Per cycle	0.722	0.964	11
No treatment after ipilimumab	0.764	Per cycle	0.666	0.998	11
Death (applied to 3 months prior to death)	0.665	Per cycle	0.532	0.798	51

TIL-NKI/CCIT: *Ex vivo*-expanded tumor infiltrating lymphocytes from autologous melanoma tumor; BRAF/MEK: v-Raf murine sarcoma viral oncogene homolog B1/mitogen activated protein kinase; PFS: Progression-free Survival; OS: Overall Survival. *Healthcare costs progressive disease: Costs for nivolumab and ipilimumab were based on the schedule 1mg/kg nivolumab + 3 mg/kg ipilimumab every 3 weeks for 4 cycles, followed by 240 mg/2weeks per model cycle until progression or death. Costs for BRAF/MEK were based on the regimen dabrafenib (150 mg/2dd)/trametinib (2 mg/1dd). Pembrolizumab was based on 200mg i.v. every 3 weeks cycle until progression or death. Temozolomide regimen was based on 150mg/m^2 twice daily for 7 days for 4 cycles and dacarbazine 850mg/m2 for 3 cycles. ±PFS and OS estimates are derived from the

TIL-NKI/CCIT-study and modelled to fit and extrapolated beyond the trial time horizon using a log-logistic distribution. ^PFS, OS and utility values are beyond 12 months and reported in more detail elsewhere. [13] A formal data request can be directed to the NKI/AvL. For terms and procedure, we refer to the data sharing agreement in the initial publication[13]

Table S2: Akaike Information Criterion (AIC) and Bayesian Information Criterion (BIC) measures used to inform best (statistical) fit of extrapolated curves using different distributions (e.g, exponential, Weibull, Gompertz, Loglogistic, and Lognormal) to expand Progression-Free Survival and Overall Survival beyond trial horizon.

TIL-NKI/CCIT				
	**Progression-Free Survival**		**Overall Survival**	
	AIC	BIC	AIC	BIC
Exponential	478.7894	481.2202	412.5095	414.9403
Weibull	471.3217	476.1833	414.4439	419.3056
**Gompertz**	**441.4333**	**446.2949**	411.3274	416.1890
Loglogistic	447.6858	452.5474	408.8950	413.7567
Lognormal	446.3604	451.2220	**405.8039**	**410.6656**
**Ipilimumab**				
	**Progression-Free Survival**		**Overall Survival**	
	AIC	BIC	AIC	BIC
Exponential	442.5565	444.9873	425.1461	427.5769
Weibull	444.4984	449.3600	427.1068	431.9684
Gompertz	435.0809	439.9426	425.5952	430.4569
**Loglogistic**	**400.2508**	**405.1125**	421.7837	426.6454
Lognormal	407.1407	412.0023	**421.2514**	**426.1130**

TIL-NKI/CCIT: *Ex vivo*-expanded tumor infiltrating lymphocytes from autologous melanoma tumor. Lower AIC and BIC measures indicate better fit, lowest measures in bold.

ten Ham RMT, *et al. J Immunother Cancer* 2024; 12:e008372. doi: 10.1136/jitc-2023-008372

Table S3: Cost input parameters in Markov decision model for scenario analysis of Denmark

	Base Case	Unit	Min	Max	Source
**Costs tumor infiltrating lymphocytes (TIL-NKI/CCIT)**					
Healthcare costs (progression-free survival)	***Base Case***	***Unit***	***Min***	***Max***	***Source***
**Screening**	**€ 6,123**	**Per patient**	**€ 4,898**	**€ 7,347**	**(11), (51)**
Physical examination	€ 5,081	Per patient	€ 4,065	€ 6,097	(11), (51)
Laboratory tests	€ 340	Per patient	€ 272	€ 408	(11), (51)
Consultations	€ 702	Per patient	€ 561	€ 842	(11), (51)
**TIL-NKI/CCIT isolation**	**€ 2,468**	**Per patient**	**€ 1,975**	**€ 2,962**	**(11), (51)**
Surgery	€ 706	Per patient	€ 565	€ 847	(11), (51)
Hospital admission	€ 1,686	Per patient	€ 1,348	€ 2,023	(11), (51)
Consultations	€ 77	Per patient	€ 61	€ 92	(11), (51)
**TIL-NKI/CCIT production**	**€ 47,931**	**Per product**	**€ 38,345**	**€ 57,518**	**(11), (51)**
**Hospital admission and follow-up**	**€ 47,908**	**Per patient**	**€ 38,326**	**€ 57,489**	**(11), (51)**
Hospital admission	€ 22,215	Per patient	€ 17,772	€ 26,658	(11), (51)
Medications	€ 13,281	Per patient	€ 10,625	€ 15,937	(11), (51)
Lab tests	€ 4,234	Per patient	€ 3,387	€ 5,080	(11), (51)
Blood products	€ 4,703	Per patient	€ 3,762	€ 5,643	(11), (51)
Consultations	€ 587	Per patient	€ 469	€ 704	(11), (51)
Others (e.g., ECG, CT, chest X-ray, supportive care)	€ 2,888	Per patient	€ 2,311	€ 3,466	(11), (51)
**Total costs of TIL-NKI/CCIT treatment**	**€ 104,430**	**Per patient**			
Health care costs (progressive disease)*	***Base Case***	***Unit***	***Min***	***Max***	***Source***
Ipilimumab monotherapy	€ 63,845	0.20	€ 34,269	€ 101,825	(11), (51, 52)
BRAF/MEK inhibitor	€ 102,036	0.20	€ 81,629	€ 122,443	(11), (51, 52)
Ipilimumab/nivolumab combination therapy	€ 71,735	0.11	€ 57,388	€ 86,082	(11), (51, 52)
Pembrolizumab	€ 56,754	0.01	€ 45,403	€ 68,105	(11), (51, 52)
No treatment	€ 0	0.43	€ 0	€ 5,000	(11), (51, 52)
Other (temozolomide, ipilimumab/pembrolizumab)	€ 91,943	0.05	€ 73,554	€ 110,331	(11), (51, 52)
Death	***Base Case***	***Unit***	***Min***	***Max***	***Source***
Costs associated with (3 months prior to) death	€ 1,577	Per patient	€ 1,262	€ 1,893	(11), (51)
Societal costs	***Base Case***	***Unit***	***Min***	***Max***	***Source***
Direct patient costs (medication, homecare, travel)	€ 302	First cycle	€ 241	€ 361	(11), (51)
Direct patient costs (medication, homecare, travel)	€ 92	>first cycle	€ 74	€ 111	(11), (51)
Direct patient costs (co pay)	€ 565	Per year	€ 452	€ 678	(11), (51)
Informal care	€ 932	First cycle	€ 746	€ 1,119	(11), (51)
Informal care	€ 130	>first cycle	€ 104	€ 155	(11), (51)
Productivity loss	€ 4,122	First cycle	€ 1,688	€ 8,548	(11), (51)
Productivity loss	€ 92	>first cycle	€ 74	€ 110	(11), (51)
**Costs ipilimumab**					
Healthcare costs (progression-free survival)	***Base Case***	***Unit***	***Min***	***Max***	***Source***
**Screening**	**€ 3,021**	**Per patient**	**€ 2,417**	**€ 3,625**	**(11), (51)**
Physical examination and lab tests	€ 3,021	Per patient	€ 2,417	€ 3,625	(11), (51)
**Ipilimumab treatment**	**€ 73,332**	**Per patient**	**€ 41,858**	**€ 113,209**	**(11), (51)**
Hospital admission	€ 2,949	Per patient	€ 2,359	€ 3,539	(11), (51)
Ipilimumab, including supportive	€ 63,845	Per patient	€ 34,269	€ 101,825	(11), (51)

ten Ham RMT, *et al. J Immunother Cancer* 2024; 12:e008372. doi: 10.1136/jitc-2023-008372

	medicines					
	Lab tests	€ 1,789	Per patient	€ 1,431	€ 2,147	(11), (51)
	Blood products	€ 329	Per patient	€ 263	€ 395	(11), (51)
	Consultations	€ 1,525	Per patient	€ 1,220	€ 1,829	(11), (51)
	Others (e.g., ECG, CT, chest X-ray, supportive care)	€ 2,895	Per patient	€ 2,316	€ 3,474	(11), (51)
	**Total costs of ipilimumab treatment**	**€ 76,353**	**Per Patient**			
Health care costs (progressive disease)*		***Base Case***	***Unit***	***Min***	***Max***	***Source***
	Ipilimumab rechallenge	€ 63,845	0.02	€ 34,269	€ 101,825	(11), (51, 52)
	BRAF/MEK inhibitor	€ 102,036	0.29	€ 81,629	€ 122,443	(11), (51, 52)
	Pembrolizumab	€ 56,754	0.10	€ 45,403	€ 68,105	(11), (51, 52)
	No treatment/other trial	€ 0	0.57	€ 0	€ 5,000	(11), (51, 52)
	Other (dacarbazine, temozolomide)	€ 7,611	0.02	€ 6,088	€ 9,133	(11), (51, 52)
Death		***Base Case***	***Unit***	***Min***	***Max***	***Source***
	Costs associated with (3 months prior to) death	€ 1,577	Per patient	€ 1,262	€ 1,893	(11), (42)
Societal costs		***Base Case***	***Unit***	***Min***	***Max***	***Source***
	Direct patient costs (medication, homecare, travel)	€ 283	First cycle	€ 241	€ 361	(11), (42)
	Direct patient costs (medication, homecare, travel)	€ 35	>first cycle	€ 28	€ 42	(11), (42)
	Direct patient costs (co pay)	€ 565	Per year	€ 452	€ 678	(11), (42)
	Informal care	€ 932	First cycle	€ 746	€ 1,119	(11), (51)
	Informal care	€ 130	>first cycle	€ 104	€ 155	(11), (51)
	Productivity loss	€ 4,122	First cycle	€ 1,688	€ 8,548	(11), (51)
	Productivity loss	€ 92	>first cycle	€ 74	€ 110	(11), (51)

TIL-NKI/CCIT: *Ex vivo*-expanded tumor infiltrating lymphocytes from autologous melanoma tumor; BRAF/MEK: v-raf murine sarcoma viral oncogene homolog B1/mitogen activated protein kinase; PFS: Progression Free Survival; OS: Overall Survival. *Healthcare costs progressive disease: Costs for nivolumab and ipilimumab were based on the schedule 1mg/kg nivolumab + 3 mg/kg ipilimumab every 3 weeks for 4 cycles, followed by 240 mg/2weeks per model cycle until progression or death. BRAF/MEK was based on regimen dabrafenib(150 mg/2dd)/trametinib(2 mg/1dd). Pembrolizumab was based on 200mg i.v. every 3 weeks cycle until progression or death. Temozolomide regimen was based on 150mg/m2 twice daily for 7 days for 4 cycles and dacarbazine 850mg/m2 for 3 cycles. ± PFS and OS estimates are derived from the TIL-study and modelled to fit and extrapolate beyond the trial time horizon using a log-logistic distribution. ^PFS, OS and utility values are beyond 12 months and reported in more detail elsewhere.(11) [13] A formal data request can be directed to the NKI/AvL. For terms and procedure, we refer to the data sharing agreement in the initial publication.(11)

Table S4: Undiscounted and discounted life years, quality adjusted life years, costs, and incremental cost-effectiveness ratios of ipilimumab compared to TIL-NKI/CCIT treatment.

	**Denmark**					
	**Undiscounted**			**Discounted^**		
	TIL-NKI/CCIT	ipilimumab	incremental	TIL-NKI/CCIT	ipilimumab	incremental
5 years						
Life years	2.46	2.11	0.35	2.32	2.00	0.32
QALYs	1.94	1.53	0.41	1.83	1.45	0.38
Costs	€213,456	€284,455	€-70,999	€204,258	€269,615	€-65,357
ICER	*dominant*			*dominant*		
10 years						
Life years	3.38	2.72	0.66	3.05	2.48	0.57
QALYs	2.64	1.99	0.67	2.40	1.81	0.59
Costs	€269,191	€360,165	€-90,974	€247,973	€329,143	€-81,170
ICER	*dominant*			*dominant*		
**Life time**						
**Total LYs**	**4.47**	**3.33**	**1.14**	**3.69**	**2.85**	**0.84**
**Total QALYs**	**3.52**	**2.46**	**1.06**	**2.91**	**2.09**	**0.81**
**Total costs**	**€337,309**	**€436,135**	**€-98,826**	**€287,587**	**€374,063**	**€-86,476**
**ICER**	***dominant***			***dominant***		

TIL-NKI/CCIT: *Ex vivo*-expanded tumor infiltrating lymphocytes from autologous melanoma tumor; QALYs: Quality adjusted life years: ICER : Incremental cost-effectiveness ratio, calculated ICER = ($Costs_{intervention}$ – $Costs_{StandardOfCare}$)/($QALY_{intervention}$ – $QALY_{StandardOfCare}$). ^Costs and benefits are discounted with 3.5% per year in line with Danish guidelines for economic evaluations.(39) Discounting is applied to adjust future costs and effects to their present value.

Figure S1: Visualization of extrapolated survival curves in comparison to trial-informed survival probability. A. Progression-Free Survival TIL (TIL-NKI/CCIT). B. Progression-Free Survival ipilimumab. C. Overall Survival TIL (TIL-NKI/CCIT). D. Overall survival ipilimumab.

A.

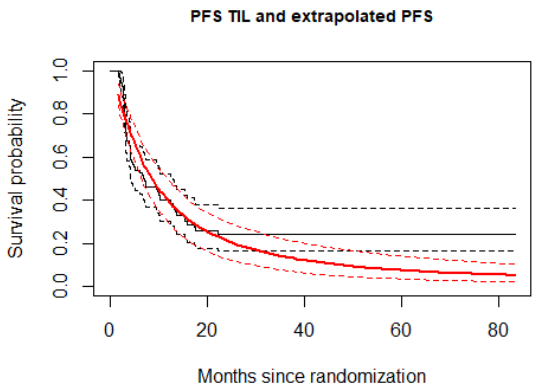

B.

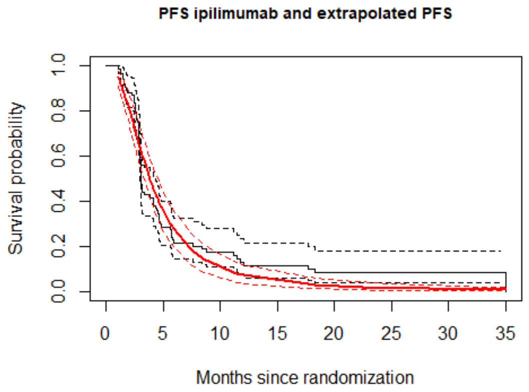

C.

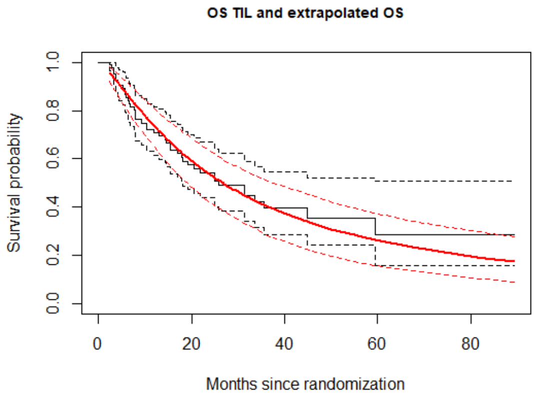

D.

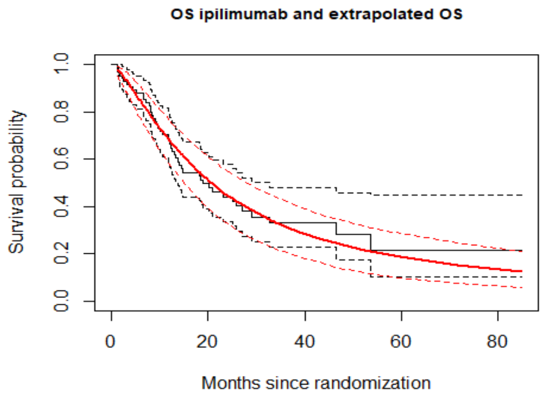

TIL (TIL-NKI/CCIT): *Ex vivo*-expanded tumor infiltrating lymphocytes from autologous melanoma tumor; PFS: Progression-Free Survival; OS: Overall Survival.(11)

Figure S2: Deterministic (univariate) sensitivity analysis of scenario 1: Denmark. Results of the deterministic (univariate) sensitivity analysis (DSA) visualized in a tornado diagram. The diagram shows impact of discounted individual parameters on the incremental cost-effectiveness ratio (ICER) by alternately varying input values one by one between pre-set minimum and maximum values (See Table 1 and S1).

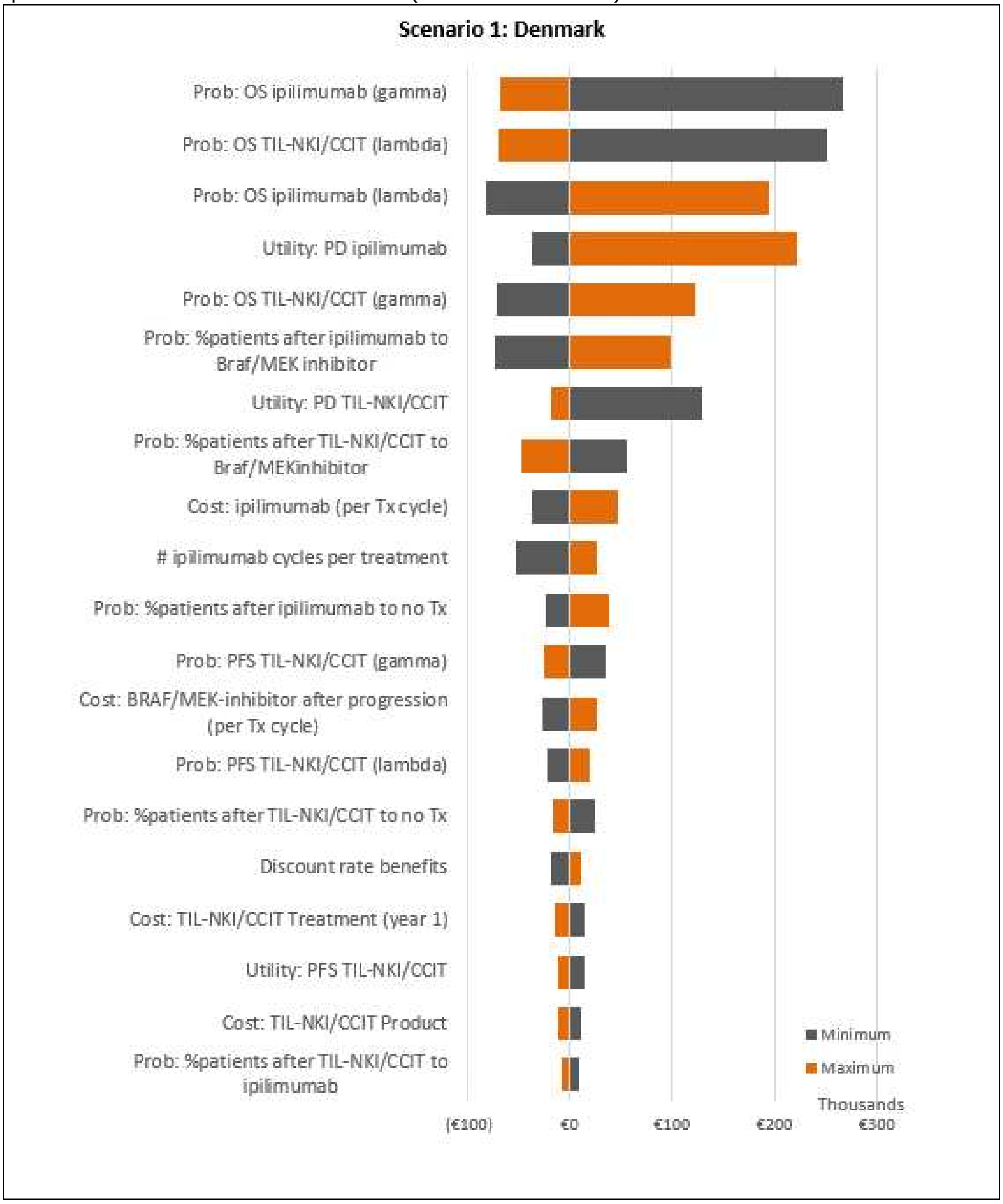

TIL-NKI/CCIT: *Ex vivo*-expanded tumor infiltrating lymphocytes from autologous melanoma tumor; ipi: ipilimumab; Prob: probability; OS: overall survival; PFS: progression-free survival; BRAF/MEK: v-raf murine sarcoma viral oncogene homolog B1/mitogen activated protein kinase; QALY: quality adjusted life year; #: number.

Figure S3: Cost-Effectiveness Acceptability Curve. Cost-effectiveness Acceptability Curve of the discounted base case showing the probability of TIL-NKI/CCIT being cost-effective given the Dutch willingness-to-pay of €80,000,- per incremental QALY gained, in 2021 euros.

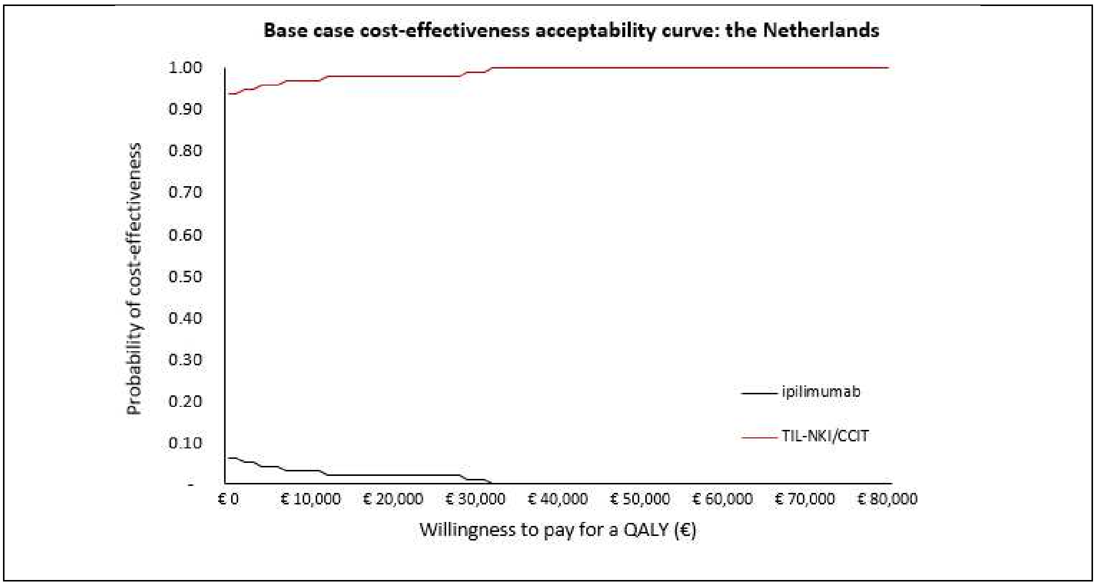

TIL-NKI/CCIT: *Ex vivo*-expanded tumor infiltrating lymphocytes from autologous melanoma tumor; QALY: quality adjusted life year.

Figure S4: Cost-Effectiveness Acceptability Curve of scenario analysis: Denmark. Cost-effectiveness Acceptability Curve of the discounted scenario showing the probability of TIL-NKI/CCIT being cost-effective given the assumed Danish willingness-to-pay of €50,000,- per incremental QALY gained, in 2021 euros.

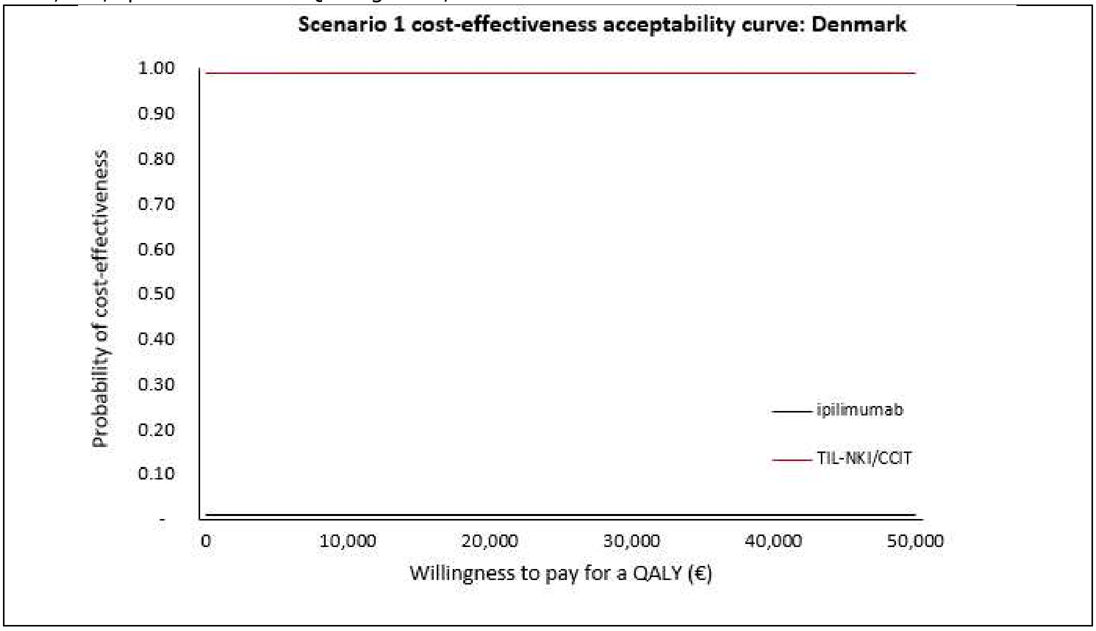

TIL-NKI/CCIT: *Ex vivo*-expanded tumor infiltrating lymphocytes from autologous melanoma tumor; QALY: quality adjusted life year.

Figure S5: Probabilistic sensitivity analyses of scenario 1: Denmark.
Results of the probabilistic sensitivity analysis (PSA) visualized in cost-effectiveness plane. The PSA shows uncertainty of estimated discounted base case incremental cost-effectiveness ratio (ICER) over a lifetime horizon by simultaneously sampling uncertainty across all parameters by 10,000 iterations. All model input parameters are sampled randomly, according to their individual appropriate distributions between pre-set minimum and maximum values (Table S2).

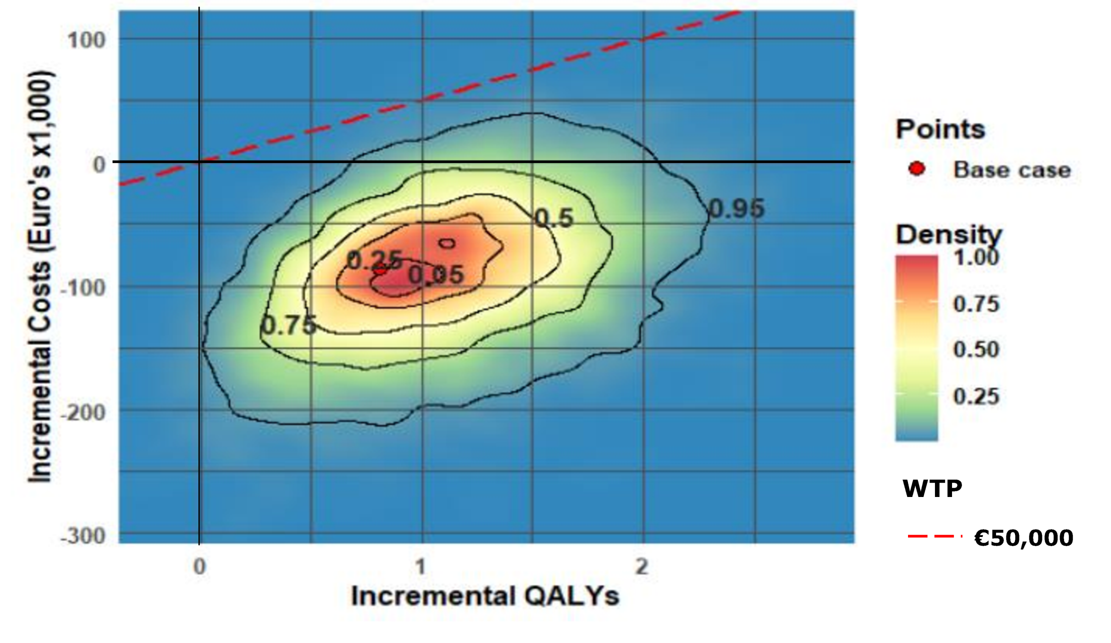

WTP: willingness to pay; QALYs: Quality adjusted life years.

Supplement to Methods: Cost estimation of TIL-NKI/CCIT production.

Costs for production of *ex vivo*-expanded tumor infiltrating lymphocytes (TIL-NKI/CCIT) from autologous melanoma tumor were estimated using a framework specifically designed to estimate manufacturing costs of cell-based therapies in small-scale and academic or public funded settings.(29) Within this framework, cost categories were defined as: material, equipment, personnel and facility. Categories are mutually exclusive to prevent double counting or overlooking of consumed resources. In addition, a distinction was made between fixed and variable costs.

Costs are considered fixed if they do not increase as the number of products or services provided increase. The sum of the fixed costs across categories (fixed material, equipment, personnel and facility costs) is considered the facility running costs and calculated per year and divided by number of TIL-NKI/CCIT batches per year. These facility running costs are consumed to ensure operability of the facility, independent of whether products are manufactured. If costs change proportionally to the quantity of delivered goods or services provided, the costs are considered variable. In estimating variable costs with increasing batch size, deployment of additional resource units were considered. This means purchase of additional pieces of equipment or the occupation of an additional clean room when maximum capacity was reached.

In identifying and allocating costs within categories, the *Costing Methodology for Hospitals* (also known as LOGEX-model) was used. This approach is depicted by the Dutch Healthcare Authority (NZa) to estimate and create insight in costs of care and associated activities in Dutch hospitals and to set prices. The same approach, however, was also applied in the Danish setting. Based on the organisational structure, fixed costs per category were allocated pro-rata to the internal divisions involved in TIL-product manufacturing. For example, when the Qualified Person within the hospital pharmacy spends 15% of its time on quality control and release of TIL-NKI/CCIT, 15% of employer expenses of this person were allocated to the fixed personnel category. Fixed materials included facility stock. Equipment and facility costs comprised annual depreciation costs, upkeep and maintenance contracts.

Variable costs included materials and equipment only used for TIL-production. Personnel directly involved in manufacturing was also allocated to the variable personnel category. In the Netherlands, manufacturing of TIL-NKI/CCIT took place approximately 50% at the Netherlands Cancer Institute (NKI), the Netherlands BioTherapeutics Unit (BTU) and 50% at Sanquin Bloodbank. In Denmark, TIL-NKI/CCIT production took place solely at the National Center for Cancer Immune Therapy (CCIT-DK), in Copenhagen.

Not included were upfront investments including research and development, building of the facility or learning effects, including product-specific training of (new) employees, product development costs, validation runs and costs associated with initial quality documentation such as Investigational Medicinal Product Dossier (IMPD) or standard operating procedures (SOP). Therefore, we emphasize that the costs estimated here are TIL-NKI/CCIT production costs only, not to be mistaken with product price.
